# Epidemic Catarrh among Horses

**Published:** 1872-10

**Authors:** 


### Editorial.

### Epidemic Catarrh among the Horses of Buffalo.

It is but a few months since our city was visited by a most fearful and fatal epidemic disease affecting the human family and confined mainly to the younger portion of it. Cerebro-spinal Meningitis was for a few weeks alarmingly prevalent and then disappeared, at least, in its epidemic form, as suddenly and mysteriously as it came. Now we have prevalent an epidemic Acute Catarrh among our horses, so sudden in its appearance and universal in its influence, as to disable, at the present writing, nine-tenths of the horse population of the city and vicinity. It is supposed upon, we think good grounds, to have traveled over from Canada, since the Provinces have been suffering from the disease for some months. The early symptom, and that which first attracts attention, is Cough, which is soon attended by free mucous secretion from the membranes of the head and throat. This secretion, in many instances, is thought to be highly acrid. The animal affected soon refuses his food, looks dull and moves reluctantly. The most intelligible name, and one which gives a physician the best idea of its nature, is epidemic catarrh, or epidemic bronchitis, as it is well known that all diseases prevailing epidemically possess a virulence rarely seen, when appearing from common causes, or as we say, sporadically. We have had occasion to acknowledge our ignorance of the causes of epidemic diseases generally, and those present with us particularly. Speculation and conjecture are rife, but nothing is known, and we are obliged to say, that the various suggestions as to the possible causes, are all of them quite unsatisfactory.

When physicians can explain or assign satisfactory cause for any epidemic, when they can trace to its source the hidden and mysterious influence, then we think this "Influenza" among horses can be traced to satisfactory cause. Its symptoms progress and termination cannot yet be fully written. It does not seem to be a very fatal malady, but is so universal in its influence as to paralyze business and leave us all on the "same *footing*". We will leave the treatment, for veterinary surgeons to determine, as we do not care to extend our therapeuties into the equine family only so far as our own favorite "nags" are concerned; these have a warm, well ventilated stable, ample food, general good care, and *no medicine.* If any of our professional friends desire to follow this example, it is open for imitation. If, however, we had a horse suffering very seriously, we should use the anodyne so universally useful in treating similar affections in men; but active, nauseating and depressing remedies will make even a horse sick.

